# New Variant of *Vibrio parahaemolyticus*, Sequence Type 3, Serotype O10:K4, China, 2020

**DOI:** 10.3201/eid2806.211871

**Published:** 2022-06

**Authors:** Yan Huang, Yue Du, Hong Wang, Dongmei Tan, Airong Su, Xiugui Li, Biao Kan, Lan Lan, Cong Qu, Bo Pang, Yunliang Shi, Mei Lin

**Affiliations:** Guangxi Zhuang Autonomous Region for Disease Control and Prevention, Nanning, China (Y. Huang, Y. Du, H. Wang, D. Tan, A. Su, X. Li, L. Lan, C. Qu, Y. Shi, M. Lin);; Guangxi Key Laboratory of Major Infectious Disease Prevention and Control and Biosafety Emergency Response, Nanning (Y. Huang, M. Lin);; State Key Laboratory of Infectious Disease Prevention and Control, Beijing, China (B. Kan, B. Pang**);**; National Institute for Communicable Disease Control and Prevention, Beijing (B. Kan, B. Pang);; School of Basic Medical Sciences of Guangxi Medical University, Nanning (Y. Shi)

**Keywords:** Vibrio parahaemolyticus, new variant, O10: K4, pandemic clone, outbreak, bacteria, China

## Abstract

In 2020, a new serotype of *Vibrio parahaemolyticus* O10:K4 emerged and caused several outbreaks and sporadic cases in Guangxi, China. Phylogenetic analysis indicated that those strains are new variants of the sequence type 3 pandemic clone. The new serotype may become dominant, warranting enhanced investigations and surveillance.

*Vibrio parahaemolyticus* is a halophilic bacterium distributed naturally in marine and estuarine environments. It is one of the most common bacterial pathogens leading to outbreaks and illness in China (*1*). In Guangxi, China, *V. parahaemolyticus* is the second most common cause of foodborne disease outbreaks.

A large proportion of the *V. parahaemolyticus* isolated during outbreaks have been O3:K6 and its serovariants, and these serovariants belonged to the pandemic clone (*2*). A total of 49 *V. parahaemolyticus* serovariants that belonged to the pandemic clone have been identified (*3*). The strains of that clone have characteristics of *tdh*+, *trh−*, *toxRS*/new+ (a unique *toxRS* sequence), and *orf8+/−* (the *orf8* sequence of f237 phage) (*2*). Furthermore, it is speculated that the appearance of derived serotypes (e.g., O4:K68, O1:K36, and O1:KUT), all of which have genetic markers and molecular profiles similar to those of the O3:K6 pandemic strains, is a selective response to host immunologic pressure of the pandemic O3:K6 serotype of *V. parahaemolyticus* (*2*,*4*).

In 2010, a laboratory-based foodborne disease surveillance system, which included municipal-level and prefecture-level monitoring laboratories, was established in Guangxi. Serotyping, pulse-field gel electrophoresis, and whole-genome sequencing are now routine methods used in this surveillance system when *V. parahaemolyticus* is isolated during outbreaks. In 2019, a total of 6 serotypes of *V. parahaemolyticus* were isolated and identified during outbreaks, and O3:K6 was predominant (68%, 42/62).

We report a new serotype of *V. parahaemolyticus*, O10:K4, which emerged in 2020 and caused infections in the Beibu Gulf area of Guangxi. O10:K4 has since become the predominant (71%, 20/28) *V. parahaemolyticus* serotype in Guangxi.

## The Study

**Figure 1:**
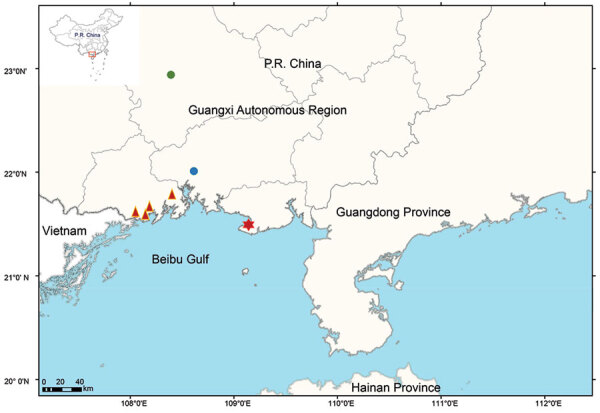
Geographic distribution of the new serotype of *Vibrio parahaemolyticus*, sequence type 3, serotype O10:K4, in Guangxi, China, 2020. Red star represents the outbreak site in Beihai; red triangles represent outbreak sites in Fangchenggang; blue circle represents the sporadic case in Qinzhou, and green circle represents the sporadic case in Nanning. Inset map shows study location in China.

In August 2020, acute gastroenteritis cases were reported in coastal cities in the Beibu Gulf area in Guangxi. In early August, 10 cases of diarrhea were reported in Beihai, a coastal city of the Beibu Gulf area (Figure 1). The patients reported fever, abdominal pain, and vomiting. All patients had consumed rice noodles in the same fast-food restaurant. We obtained 7 *V. parahaemolyticus* isolates from the patients and 1 strain from a sample of instant sour bean (nonseafood) in the restaurant. Slide agglutination of the 8 *V. parahaemolyticus* isolates showed presence of the O10:K4 serotype.

At the end of August, ≈120 cases of acute gastroenteritis were reported in Fangchenggang, another coastal city in the Beibu Gulf area. Those patients also reported fever, abdominal pain, nausea, and vomiting. Ten strains of serotype O10:K4 *V. parahaemolyticus* were obtained from hospitalized patients. The investigation indicated that no food had been shared by the patients, although all had consumed durians before symptom onset. The durians that these patients consumed had all been accidentally soaked in seawater. We speculate that those durians were contaminated with *V. parahaemolyticus* and that their consumption might have contributed to the infections. However, we could not isolate serotype O10:K4 *V. parahaemolyticus* from the same batch of durians that the patients consumed, although we obtained other serotype strains (O4:K13, O1:K25, O1:K33, O3:Kunk, and O4:Kunk). Follow-up surveillance detected 2 more strains of O10:K4 isolated from diarrhea patients in Qinzhou (another coastal city, on October 20, 2020) and Nanning (an inland city >200 km from the sea, on November 15, 2020) (Figure 1).

To explore the genetic position of these 20 O10:K4 isolates from persons in 4 cities, we performed whole-genome sequencing on a MiSeq platform (Illumina, https://www.illumina.com). We assembled whole-genome sequences de novo by using SPAdes v.3.12.0 (*5*) (GenBank accession nos. JAHWYL000000000, JAKJNF000000000–JAKJNW000000000) and subtyped them by using in silico multilocus sequence typing on PubMLST (https://pubmlst.org/organisms/vibrio-parahaemolyticus). All strains belonged to sequence type (ST) 3 and clonal complex 3, which is the sequence profile for most pandemic strains of *V. parahaemolyticus.*

**Figure 2:**
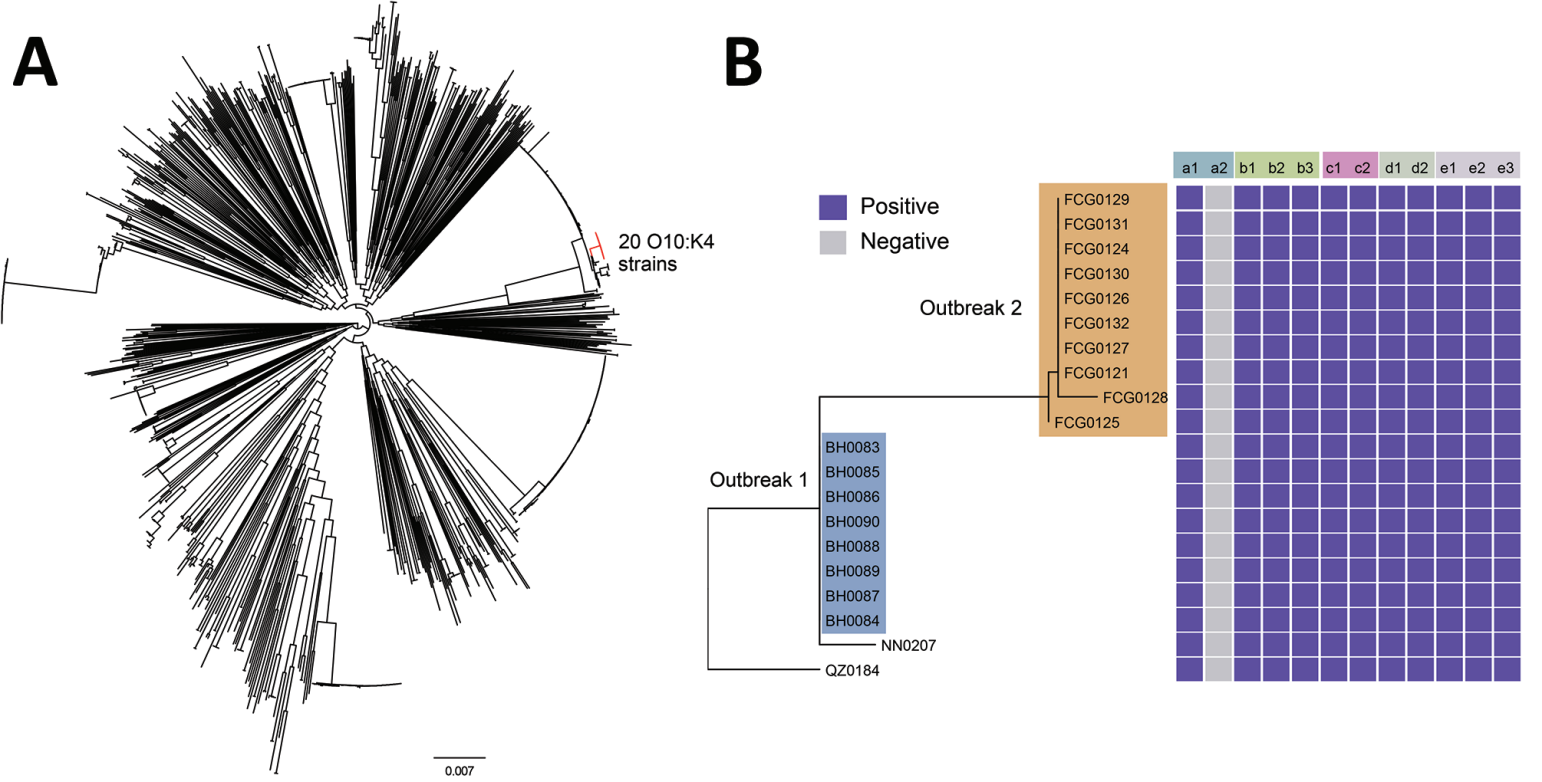
Phylogenetic tree based on the single-nucleotide variations in the core genomes of 1,120 *Vibrio parahaemolyticus* genomes: 20 isolates from patients in the Beibu Gulf area of Guangxi, China, 33 isolates collected in Guangxi in recent years, and all 1,067 genomic sequences available in the PubMLST database (https://pubmlst.org/organisms/vibrio-parahaemolyticus) (Appendix). A) Maximum-likelihood tree based on the single-nucleotide variations in the nonrepetitive, nonrecombinant regions of the genomes. Branches in red indicate the O10:K4 serotype strains. Scale bar indicates frequency of single-nucleotide variations. B) Distribution of virulence genes, pathogenic islands, secretion systems, characteristic genes in pandemic clones, and antimicrobial resistance genes. a1, *tdh*; a2, *trh*; b1, VPaI-2; b2, VPaI-3; b3, VPaI-4; c1, T3SS; c2, T6SS1; d1, *orf8*; d2, *toxRS*/new; e1, tet(34); e2, tet(35); e3, *blaCARB*-22.

We then integrated those genomic data with 33 various serotypes of *V. parahaemolyticus* isolated in Guangxi in recent years, as well as all 1,067 *V. parahaemolyticus* genomic sequences available in the PubMLST database (through January 14, 2022) (*6*) (additional *V. parahaemolyticus* phylogenetic information in Appendix). We constructed a maximum-likelihood tree based on the single-nucleotide variations (SNVs) identified in the nonrepetitive and nonrecombinant core genome (Figure 2, panel A). The O10:K4 *V. parahaemolyticus* formed a unique, exclusive, and tight cluster that was most closely related to a strain isolated in China in 2016 (strain VP161407), which was also ST3. This O10:K4 cluster is part of the ST3 clade. 

We next focused on the 20 O10:K4 strains and strain VP161407. We reconstructed a maximum-likelihood tree based on the SNVs determined in the core genomes of these 21 strains. We found that strain QZ0184, isolated in Qinzhou, was most closely related to strain VP161407. To further investigate the relationship between the 20 O10:K4 strains in detail, we reconstructed a maximum-likelihood tree based on the SNVs in the core genomes of the 20 strains. We found that strains isolated in Beihai and Fangchenggang formed 2 separate clusters, which indicated 2 independent outbreaks. We then detected virulence genes, pathogenic islands, and antimicrobial resistance genes in the O10:K4 strains. Analysis revealed that the characteristic genes in these O10:K4 strains were same as those in the *V. parahaemolyticus* pandemic clone: *tdh+*, *trh*–, *toxRS*/new+, and *orf8+* (Figure 2, panel B). We also detected type 3 and type 6 secretion systems, VPaI-2, VPaI-3, and VPaI-4 in those strains (Figure 2, panel B). Moreover, we detected 3 antimicrobial resistance genes: *tet*(34), *tet*(35), and *bla_CARB_*_-22_ (Figure 2, panel B). 

## Conclusions

The new variant of ST3 *V. parahaemolyticus* O10:K4 exhibited characteristics of the *V. parahaemolyticus* pandemic clone and caused outbreaks in the Beibu Gulf area. More recently, this variant led to cases in Nanning, which indicated transmission of this variant of *V. parahaemolyticus* from coastal areas to inland areas. The variant was also detected in several other provinces in China, which indicated its widespread nature (B. Pang, unpub. data). The emergence of serotype O10:K4 may be the response to host immunologic pressure, which was observed in serotype O4:K68 (*2*,*4*). The Beibu Gulf is also known as the Gulf of Tonkin, and Vietnam is located to its west. Therefore, similar to what was observed in a previous cholera study (*7*), the possibility remains that this variant has been circulating in the Beibu Gulf area, over time leading to infections in the countries around it.

Appendix Supplemental information for phylogenetic analysis of *Vibrio parahaemolyticus*.
